# Mechanisms of robustness in gene regulatory networks involved in neural development

**DOI:** 10.3389/fnmol.2023.1114015

**Published:** 2023-02-06

**Authors:** Camila D. Arcuschin, Marina Pinkasz, Ignacio E. Schor

**Affiliations:** ^1^Instituto de Fisiología, Biología Molecular y Neurociencias (IFIBYNE), Universidad de Buenos Aires—Consejo Nacional de Investigaciones Científicas y Técnicas (CONICET), Buenos Aires, Argentina; ^2^Departamento de Fisiología, Biología Molecular y Celular, Facultad de Ciencias Exactas y Naturales, Universidad de Buenos Aires, Buenos Aires, Argentina

**Keywords:** robustness, neuronal differentiation, transcriptional regulation, development, regulatory elements, gene regulatory networks, genetic variation

## Abstract

The functions of living organisms are affected by different kinds of perturbation, both internal and external, which in many cases have functional effects and phenotypic impact. The effects of these perturbations become particularly relevant for multicellular organisms with complex body patterns and cell type heterogeneity, where transcriptional programs controlled by gene regulatory networks determine, for example, the cell fate during embryonic development. Therefore, an essential aspect of development in these organisms is the ability to maintain the functionality of their genetic developmental programs even in the presence of genetic variation, changing environmental conditions and biochemical noise, a property commonly termed robustness. We discuss the implication of different molecular mechanisms of robustness involved in neurodevelopment, which is characterized by the interplay of many developmental programs at a molecular, cellular and systemic level. We specifically focus on processes affecting the function of gene regulatory networks, encompassing transcriptional regulatory elements and post-transcriptional processes such as miRNA-based regulation, but also higher order regulatory organization, such as gene network topology. We also present cases where impairment of robustness mechanisms can be associated with neurodevelopmental disorders, as well as reasons why understanding these mechanisms should represent an important part of the study of gene regulatory networks driving neural development.

## Introduction

In the development of multicellular organisms, gene regulatory networks (GRN) that determine cell fate and drive differentiation must be resilient to genetic, environmental or random perturbations, including changing temperature, variable number of embryonic cell progeny, gene expression noise, retrotransposon insertions, epigenetic constraint relaxation, and somatic or germline point mutations. Decades ago, Waddington introduced the idea of canalized development (Waddington, 1942) and from there on the concept of robustness has emerged as a key feature of biological processes that favors a uniform outcome (phenotype) in the presence of variable conditions (Barkai and Shilo, 2007; Félix and Wagner, 2008). The importance of robustness during organisms development and its impact on evolution, as well as several examples across different models has been already extensively reviewed before (see for example Scharloo, 1991; Kirschner and Gerhart, 1998; de Visser et al., 2003; Félix and Wagner, 2008; Payne and Wagner, 2015). Here, we aim to present examples of robustness mechanisms assisting gene expression regulation necessary for neural development.

## Assessing robustness of developmental processes

Proper development is crucial for survival of multicellular organisms. Since this process faces perturbations in the form of high rates of genetic variation (Keightley, 2012), environmental changing conditions or even stochastic noise, it is not surprising that developmental robustness mechanisms have appeared across the evolution. But before discussing robustness mechanisms, it is important to clarify how robustness can be identified.

In its simplest conceptual meaning, robustness implies the persistence of a phenotype in the face of perturbation. However, considering the mentioned sources of perturbations to which each individual is exposed, robustness can be associated, as suggested by Félix and Wagner, with the lack of phenotype variability amongst a population (Félix and Wagner, 2008). This association originates in some of the first examples in which developmental robustness has been studied, such as bristle number and *ocelli* with bristles in *Drosophila*, and *vibrissae* number in mice (Scharloo, 1991). However, not all biological traits need to be robust and robustness does not always mean lack of variability at all levels of analysis (Hiesinger and Hassan, 2018). In nature, some phenotypes may seem to lack robustness and show variability between individuals because there is no selective pressure acting on that variable phenotype. But sometimes, some level of variability is actively used to acquire a specific output, as observed for example in the variability of cell-to-cell expression of Dscam1 isoforms which regulates self-avoidance in *Drosophila melanogaster* mushroom bodies (Kise and Schmucker, 2013; Lawrence Zipursky and Grueber, 2013).

Following Waddington's idea of canalization (Waddington, 1942), we normally see robustness of developmental processes buffering minor variations, such as noisy gene expression (Eldar et al., 2002; Arias and Hayward, 2006; Urban and Johnston, 2018) or disruption of single regulatory elements (Kvon et al., 2021), while larger perturbations (such as complete ablation of a gene) could be able to override most robustness mechanisms. In this review we will focus on mechanisms affecting regulation of gene expression and GRN function, although many molecular and cellular features are able to buffer phenotypes against perturbations, including exploratory behavior (Sperry, 1963; Kirschner and Gerhart, 1998; Wit and Hiesinger, 2023), progenity compensation between lineages (Enriquez et al., 2018), chaperones-target interactions (Sato, 2018), or weak linkage of protein interactions in cell signaling (Kirschner and Gerhart, 1998; Hartman et al., 2001).

## The nervous system as a developmental model

The majority of adult neural cell diversity is generated in the embryonic and early postnatal stages in mammals, and larval stages in *Drosophila*, from a pool of undifferentiated neural stem and progenitor cells (Mira and Morante, 2020). Neural stem cells are multipotent and generate the main cell types of the nervous system: neurons, oligodendrocytes, and astrocytes. Typically, stem cells initially generate neurons and afterwards glial cells, and this switch from neurogenesis to gliogenesis requires changes in stem cell properties that are dependent on extrinsic and intrinsic factors (Qian et al., 2000; Temple, 2001; Ohtsuka and Kageyama, 2019; Villalba et al., 2021). Many signaling pathways are known to regulate this switch in cell specification (Perrimon et al., 2012; Maury et al., 2015). For example in mammals, signals such as Bone morphogenetic protein 2 (BMP2) and erythropoietin (Epo) induce proneural gene expression (Bertrand et al., 2002). To prevent other cells from differentiating to neurons, Notch signaling downregulates proneural genes (Lowell et al., 2006; Lathia et al., 2008; Sjöqvist and Andersson, 2019; Bocci et al., 2020). These pathways maintain a balance between progenitors entering a neuron differentiation pathway and progenitors remaining undifferentiated and available to produce other types of nervous system cells (Bertrand et al., 2002). In addition to this general neural differentiation program gradients of morphogens, molecules secreted by specific sources that can diffuse through the tissue, determine cell fate along specific axes. For example, in the development of the neural tube, a gradient of Sonic Hedgehog (Shh) control cell types along the ventral-dorsal axis. Interestingly, the Shh gradient is able to create boundaries that define cell type in a very robust manner (Hernandez-Miranda et al., 2017; Sagner and Briscoe, 2017, 2019; Xia et al., 2022). This process uses Shh concentration along time and space as input, and depends on incoherent feedforward (Mangan and Alon, 2003) and feedback loops that connect Shh signaling with the expression of the Olig2, Nkx2.2, and Pax6 transcriptional regulators (Balaskas et al., 2012).

While most neurogenesis in mammals occurs during development or very early in the newborn, there has been an increased interest in the past years in adult neurogenesis (Ernst et al., 2014; Falk and Götz, 2017; Denoth-Lippuner and Jessberger, 2021). New neurons in the adult can contribute to normal pattern separation, cognition and learning (Clelland et al., 2009; Sahay et al., 2011; Nakashiba et al., 2012) and it has been shown a reduction of this process in patients with neurodegenerative disease such as Alzheimer's (Moreno-Jiménez et al., 2019; Tobin et al., 2019). However, the presence of newborn neurons in the human adult hippocampus has been recently disputed (Paredes et al., 2018; Sorrells et al., 2018; Alvarez-Buylla et al., 2022). Although this issue remains controversial, elucidating mechanisms that ensure the robustness in neural development might be relevant also in the adult brain.

In summary, nervous system development in metazoans is a highly complex process influenced by many factors that converge on interconnected gene regulatory networks (GRNs) dictating specific spatio-temporal differentiation patterns of cells. It is therefore expected, as many other developmental processes, to be sensitive to perturbations, both from the cellular environment and the external environment (McGrath et al., 2011), which suggests that some of the key steps in this process might exhibit robustness mechanisms. The idea of robustness in neurodevelopment is supported by genetic evidence, such as that even in the presence of high levels of inter-individual genetic variation (Keightley, 2012), the human neurodevelopmental transcriptome is much more robust across individuals than across time or regions (Silbereis et al., 2016).

## Mechanisms of robustness in developmental gene regulatory networks

Over the years, evidence of different mechanisms that give robustness to embryonic development has accumulated (Arias and Hayward, 2006; Barkai and Shilo, 2007; Félix and Wagner, 2008; Rogers and Schier, 2011; Payne and Wagner, 2015), although not so much is known in particular for the development of the nervous system, specially in vertebrates. Nevertheless, we will present some examples affecting gene expression programs involved in development, to illustrate how these mechanisms operate.

### Robustness at the level of individual gene expression

Even though development and differentiation are controlled by GRNs consisting of several genes, these networks have hierarchies, implying that reduced expression variability for some specific highly connected genes might be advantageous for the regulatory function of the network as a whole. For example, it has been identified that in many gene regulatory networks there are genes that act as master regulators (Carro et al., 2010; Tutukova et al., 2021), corresponding to regulatory bottlenecks. Therefore, we will present examples of mechanisms that control robustness in the regulation of gene expression, both at the transcriptional and post-transcriptional levels.

The most basic form in which transcription of a certain gene can be robust to, for example, a genetic perturbation is the fact that most individual transcription factors (TFs) are typically able to bind to many different sequences, which are connected to each other in the “genotype space” (Badis et al., 2009; Payne and Wagner, 2014, 2015). This allows TF binding conservation in the presence of binding site variation. In addition, commonly regulated binding sites tend to be spatially clustered in the genome within regulatory elements (Berman et al., 2002), providing a further level of robustness to binding site turnover. While TF redundancy provides robustness on the function of an individual enhancer, the presence of redundant enhancer regions have been also shown to act as a buffer against perturbations (Frankel et al., 2010; Kvon et al., 2021). Genes involved in development are enriched in more than one enhancer whereas housekeeping genes are usually controlled by a single regulatory region (Cannavò et al., 2016; Kvon et al., 2021), and redundant enhancers are depleted in GWAS-or eQTL-associated SNPs compared to single enhancers (Song and Ovcharenko, 2018), suggesting a lower impact of genetic variation in these genomic regions. Several genes with key roles in neuronal development seem to have a regulatory architecture with redundant enhancers. For example, redundant enhancers were identified controlling the expression of Shh in the ventral spinal cord, hindbrain, and telencephalon (Jeong et al., 2006). In zebrafish, six elements were described to regulate Krox20 expression in the hindbrain. Krox20 is a transcription factor important for hindbrain segmentation and patterning and highly conserved in vertebrate evolution. These elements were found to act redundantly at some extent, since while deletion of one element leads to a mild reduction in expression, deletion of two regulatory elements is needed to see a drastic impairment of Krox20 expression (Torbey et al., 2018). In mice, removal of an enhancer region located upstream of the promoter for Pax3, a transcription factor required for normal neural crest development, was insufficient to inhibit neural crest expression of Pax3 and resulted in a viable mouse. This observation led to the identification of a functionally redundant intronic enhancer that might be involved in robustness of Pax3 expression in the developing neural crest (Degenhardt et al., 2010). In addition to the enhancer configuration, the presence of redundancy at the TF level, for example due to gene duplication, can also act as a robustness mechanism in development. One example in the nervous system is the partially redundant role of the Gsx1 and Gsx2 TFs in the control of neuronal vs. glial differentiation of neuronal progenitors in the ventral telencephalon of mice (Chapman et al., 2018).

Post transcriptional mechanisms also contribute to robustness in neural development. Micro RNAs (miRNAs) in particular have been proposed as gene expression buffers in regulatory networks and are usually involved in regulatory feedback and feedforward loops (Tsang et al., 2007; Ebert and Sharp, 2012; Ghosh et al., 2014). One example of a miRNA-based post-transcriptional mechanism of developmental process robustness is the regulation of cyclin D1 expression by miR-20a/b and miR-23a in mouse cortical neurogenesis. Cyclin D1 induces expression of miR-20a/b and represses miR-23a in a feedback regulatory network. When any of these miRNAs are inhibited, the variance and the mean expression of cyclin D1 protein in progenitors increases, reducing neuronal differentiation (Ghosh et al., 2014). In another example, *Drosophila*'s miR-9a reduction and consequent dysregulation of the *senseless* transcription factor make cell phenotype more sensitive to genomic and environmental variation (Cassidy et al., 2013). Interestingly, miR-9a is conserved at the sequence level from flies to humans, suggesting that it may have a similar role in mammalian neurogenesis (Li et al., 2006).

Finally, we want to present two other elements that have lately been proposed as having a role in gene expression robustness: chromatin conformation and the promoter architecture. The organization of the genome in topologically associating domains (TADs) impacts the regulatory landscape of mammalian genomes. TAD boundaries, typically formed in regions containing clusters of CTCF binding sites, are important to instruct interactions between regulatory elements, such as enhancers and promoters, and the TAD organization has been proposed to bring robustness and precision to gene expression in development (Despang et al., 2019; Anania et al., 2022). Promoter-enhancer interactions can be also aided by CTCF-mediated chromatin loops, increasing robustness of enhancer regulation on gene expression. For example, a recent study (Paliou et al., 2019) found that loss of the interaction between the Shh gene promoter and a distal enhancer through the deletion of CTCF binding sites causes a mild decrease of Shh expression and no phenotypic change by itself, but sensitizes Shh expression to partial disruptions of the distal enhancer. Regarding the influence of the gene promoter, evidence from population genomics for both vertebrates and invertebrates strongly suggests that certain promoter architectures are associated with higher genetic robustness, perhaps buffering levels of gene expression against the effects of genetic variation (Schor et al., 2017; Sigalova et al., 2020; Einarsson et al., 2022). The relevance of these features in the development of the nervous system remains yet to be assessed.

### Robustness at higher levels of complexity

**Figure 1:**
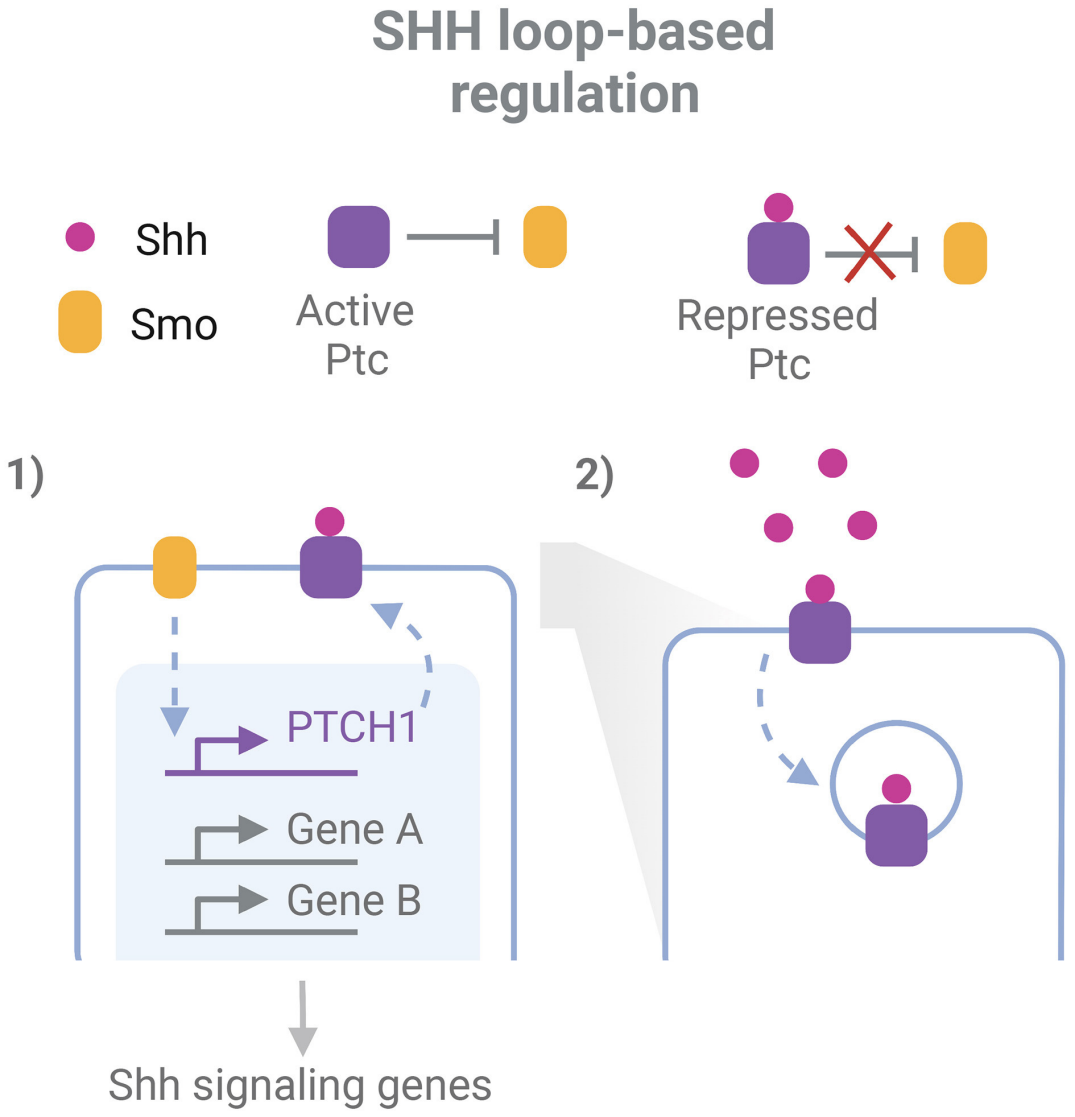
Example of network-level robustness for the action of a morphogen in neurodevelopment. Two feed-back loops participate in the robustness mechanism for the cellular responde to Shh in the neural tube: 1. Smo indirectly induces transcription of the PTCH1 gene. This gene codes for the protein Ptc, which represses Smo activity and is inhibited by Shh binding; 2. After Shh binding, Ptc mediates its endocytosis and degradation. Inspired in Sagner and Briscoe (2017) and Ferent et al. (2019). Created with BioRender.com.

While robustness exists for the expression of individual key regulators of a process, some robustness features arise when the GRN and their related signaling pathways are considered (Félix and Wagner, 2008). The topology of developmental regulatory pathways is characterized by the extensive interplay between them, the presence of feedback and feedforward loops and the redundant outputs that bring robustness to perturbations (Arias and Hayward, 2006; Barkai and Shilo, 2009). Morphogen gradients acting in the neural tube development display a great precision not only in their space and time specific extracellular levels but even more in their intracellular expression (Vetter and Iber, 2022). Regulatory loops are in part responsible for these properties, and at the same time seem to account for tolerance to perturbations of morphogen systems (Barkai and Shilo, 2009; Irons et al., 2010; Rogers and Schier, 2011). These loops are evident, for example, when analyzing the canonical Shh response (Figure 1). Shh binds to the receptor Ptc, inhibiting its repressive function over Smo, another membrane protein that subsequently triggers Shh signaling pathways. These pathways result in the activation of Gli transcription factors (TF), which drive chromatin remodeling and transcriptional regulation at different regulatory elements along the ventral-dorsal axis, inducing transcription of specific target genes (Vokes et al., 2008; Oosterveen et al., 2012; Delás et al., 2022). Remarkably, this includes a feedback loop through the upregulation of the PTCH1 gene, which codes for Ptc, resulting in an Shh-induced increase of Ptc levels (Goodrich et al., 1996; Marigo and Tabin, 1996; Sagner and Briscoe, 2017). Since the receptor has the ability also to endocyte and degrade Shh, this system also acts as a negative feedback loop to buffer variations in Shh levels and prevent its diffusion to further regions (Chen and Struhl, 1996; Dessaud et al., 2007; Rogers and Schier, 2011; Ferent et al., 2019). In addition, the response to Shh is mediated by a network of interconnected TFs downstream Gli, including Pax6, Olig2, and Nkx2.2, which plays important roles in the specific transcriptional responses and the adaptation of cells to variable spatio-temporal Shh concentration, while at the same time provides robustness against transient variation of the GRN effectors (Dessaud et al., 2010; Balaskas et al., 2012).

In the previous cases we evaluate robustness at the molecular and cellular level. However, for many complex phenotypes, variability at these levels can be compensated by mechanisms at a higher order. For example, the total number of astrocytes in the fly thorax neuropil is robust to variations in the astrocytes produced by the main precursor lineage, because secondary lineages may compensate for variability through an unknown plastic mechanism (Enriquez et al., 2018). Thus, in many cases it is important to study variability and compensation at different levels of organization (molecular—cellular—cell populations—system) to fully understand robustness of a specific phenotype.

## Developmental disorders and robustness to genetic variation

During neurodevelopment, disruption of robustness mechanisms at different levels or excessive perturbation levels that overtake them can lead to developmental disorders. For example, regarding miRNA-based feedback mechanisms, haploinsufficiency of the miR-17/92 cluster is found in some cases of Feingold syndrome, a disease that affects development and produces, among other phenotypes, microcephaly. Moreover, an hemizygous deletion of the cluster causes related symptomatology in mice (de Pontual et al., 2011). Even gene redundancy can be insufficient to buffer the effect of some mutations; while the two related tubulin genes TUBB2A and TUBB2B can partially compensate the loss of each other on their role in neuronal development, missense mutation causing gain-of-function phenotypes for one of these genes can trigger aberrant behavior unable to be counteracted by the paralogue, and this can lead to cortical malformations (Bittermann et al., 2019). For heterozygous loss-of-function mutations in X-linked genes, there might be a cross-over between the expression of the mutated allele and a skewness in the X-chromosome inactivation (XCI) process. Rett syndrome, which causes dementia, seizures and microcephaly among other phenotypes, is associated with mutations in the X-linked gene coding for MeCP2, a transcriptional regulator that binds to 5 hmC in regulatory regions of neurodevelopmental genes (Jang et al., 2017). Typically, since XCI occurs randomly in each cell, heterozygous mutant females are mosaic, and most of them actually show mild or no symptoms. However, there is evidence that in some cases of MeCP2 mutations an unbalanced XCI is seen in favor to the wild-type allele as well as a selective growth advantage of wild-type expressing cells over mutated ones (Dragich et al., 2000; Young and Zoghbi, 2004; Chahrour and Zoghbi, 2007). In other scenarios, disease symptoms will appear after a threshold of perturbation is exceeded, suggesting a limit for effectiveness of robustness mechanisms. For example, the number of CGG repeats expansion on the 5' UTR of the FMR1 gene is clearly related to the probability of having Fragile X Syndrome mental disorder. While normal individuals have between 6 and 40 CGG repeats, and in premutation cases this number can increase between 55 and 199, full-mutation cases that show disease symptoms will show a number of repeats in the range 200–230 (Nolin et al., 1996; Kronquist et al., 2008).

Of particular relevance to human health, common genetic variation is a pervasive source of perturbations for the regulatory systems of biological processes. Common variation can be seen as small-effect perturbations, which by itself are not enough to significantly affect high-order phenotypes. However, they can interact additively or non-additively with other perturbations, for example contributing to a higher chance of having a particular disease. In particular, we use the term cryptic genetic variation to define common variants that are potential disruptors of the normal expression patterns but do normally not affect phenotypes observed in a population due to, for example, epistatic interactions with other variants (Gibson and Dworkin, 2004; Gibson, 2009), allowing them to persist in the population due to lack of sufficient purifying selection. Over the last years there has been a growing interest to elucidate how common variation can shape diseases or developmental disorders using, for instance, genome wide association studies (GWAS). Most variants identified by GWAS studies are in non-coding regions, suggesting that in most cases functional common variation affects gene expression regulation (Edwards et al., 2013). While having small effects on high-level phenotypes, the high number and frequency in the population of these common variants make them an important contributor to human disease. In ASD and other neuropsychiatric disorders, for example, accountability for genetic risk is more likely to reside in common variation (Gaugler et al., 2014; Autism Spectrum Disorder Working Group of the Psychiatric Genomics Consortium and Ripke, 2019), and genes involved in corticogenesis seem to be enriched in ASD-linked common variation (Parikshak et al., 2015; Autism Spectrum Disorder Working Group of the Psychiatric Genomics Consortium and Ripke, 2019).

## Discussion

**Figure 2:**
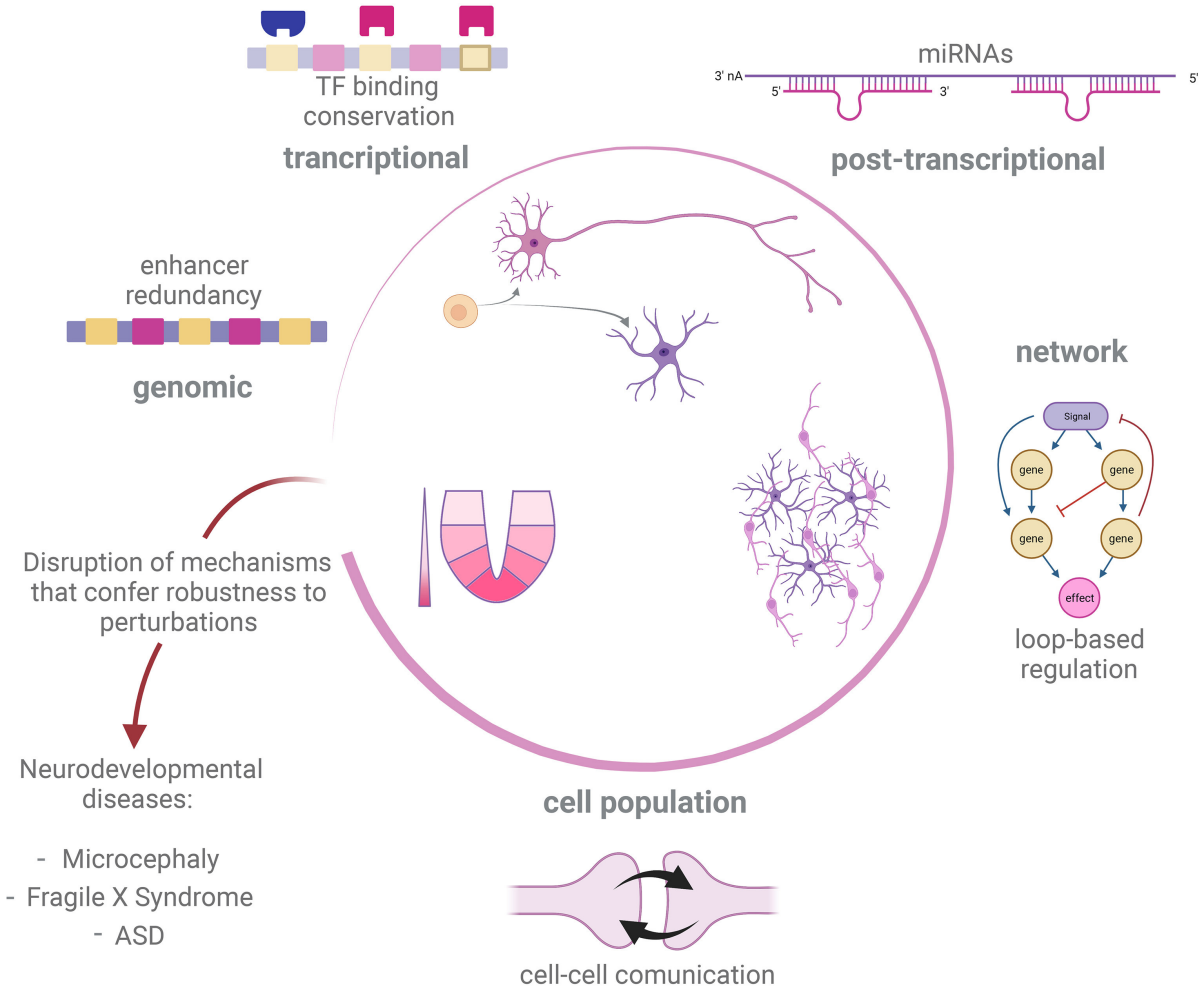
Robustness mechanism underlying development of nervous system. Development of the nervous system in different metazoan species is tuned by distinct but overlapping regulatory processes, such as sensing of morphogen gradients, cell-cell communication and GRN governing cell fate decisions. In order to correctly deliver such a complex output, different strategies promote robustness against internal or external perturbations at different levels of regulation. Disruption of robustness mechanisms could impair success of some of these developmental processes and therefore trigger neurodevelopmental disease and abnormal conditions, such as microcephaly, Fragile X Syndrome and ASD. Created with BioRender.com.

We have shown possible mechanisms at different levels of organization by which regulation of developmental processes in the nervous system can be robust to genetic and non-genetic perturbations (Figure 2). In many cases, the mechanisms of developmental robustness have been studied in relatively simple model organisms, such as *C. elegans* or *D. melanogaster*, while the study of robustness is still an underdeveloped aspect in the field of vertebrate neurodevelopment, perhaps with the exception of morphogens action (Barkai and Shilo, 2009; Irons et al., 2010; Balaskas et al., 2012; Perrimon et al., 2012; Vetter and Iber, 2022). In the present text we have presented examples of how perturbation of developmental GRNs can give rise to atypical developmental conditions and disease. We therefore believe that a deeper understanding of mechanisms that give robustness to nervous system development is needed to fully understand the genetic aspects of disorders affecting this process.

Regarding how robustness mechanisms originate, it is still an open question whether they can be heritable traits subjected to natural selection, as it is uncertain to what extent the mechanisms contributing to neurodevelopment have arised by evolutionary refinement across history. Some evidences question this idea of developmental refining, at least in human evolution, postulating that hominid brain is particularly vulnerable to perturbations in part because its great expansion in such a short evolutionary time couldn't have allowed the evolution of robust developmental trajectories (McGrath et al., 2011). In this sense, there is a significant need to test neurodevelopmental robustness mechanisms in different mammalian species to address if their role could be ancestral or if robustness of GRN involved in neuronal development has evolved repeatedly through convergence from other pre-existing mechanisms (Conant and Wagner, 2003).

## Author contributions

All authors have made a substantial and direct contribution to the work, including writing, reviewing and editing of the manuscript, and approved it for publication.
